# Biphasic Peritoneal Mesothelioma: A Lethal Clinical Entity

**DOI:** 10.7759/cureus.22638

**Published:** 2022-02-26

**Authors:** Jill David, Abdul Waheed, Shahin Foroutan, Audrey McCloskey, Harmanprit Randhawa, Frederick D Cason

**Affiliations:** 1 General Surgery, HCA Healthcare/University of South Florida Morsani College of Medicine GME/Regional Medical Center Bayonet Point Program, Hudson, Florida, USA; 2 Surgery, San Jaoquin General Hospital, San Jaoquin, USA; 3 General Surgery/Trauma and Critical Care, San Joaquin General Hospital, French Camp, USA; 4 Surgery, St. George's University School of Medicine, St. Georges, GRD; 5 Emergency Medicine, San Joaquin General Hospital, French Camp, USA; 6 Surgery, San Joaquin General Hospital, French Camp, USA

**Keywords:** neoplasms, abdominal, mesotheliomas, peritoneal, biphasic

## Abstract

Mesotheliomas are a rare malignancy of the serosal membrane. Mainly it affects the pleural surfaces followed by the second most common location, “peritoneum.” The disease follows an aggressive pattern of spread, and by the time the diagnosis is established, the condition significantly spreads to distant locations. Diagnosis of malignant peritoneal mesothelioma is typically made by tissue biopsy. The standard treatment is radical resection; however, patients have benefited from several other modalities. The current case report describes a unique case of malignant mesothelioma, biphasic peritoneal mesothelioma (BPM), which comprises less than 25% of all peritoneal mesotheliomas. The diagnosis and treatment do not differ from other subtypes; however, the prognosis is poor, and if untreated, the survival is typically less than six months.

## Introduction

Malignant mesotheliomas are rare and aggressive tumors arising from serous linings of the pleura (65%-70%), peritoneum (30%), tunica vaginalis testis, and pericardium (1%-2%) [1]. Peritoneal mesothelioma (PM) was first described by Miller and Wynn in 1908 [2]. Histologically, PM is classified into three subtypes: epithelioid, sarcomatoid, and biphasic [2]. A biphasic tumor is defined as having both epithelioid and sarcomatoid components [3]. The diagnosis of malignant peritoneal mesothelioma requires biopsy; however, diagnosis is often delayed due to the nonspecific clinical presentation, resulting in a more advanced form of this tumor [3].

The recommended treatment for patients with confirmed malignant peritoneal mesothelioma is radical resection [4]. Other treatment modalities include intensive loco-regional therapeutic strategies: cytoreductive surgery (CRS), hyperthermic intraperitoneal chemotherapy (HIPEC), and immunotherapy [5]. We present the case of a 70-year-old female patient with right upper quadrant (RUQ) abdominal pain, nausea, malaise, and unintentional weight loss secondary to biphasic peritoneal mesothelioma (BPM).

## Case presentation

**Figure 1:**
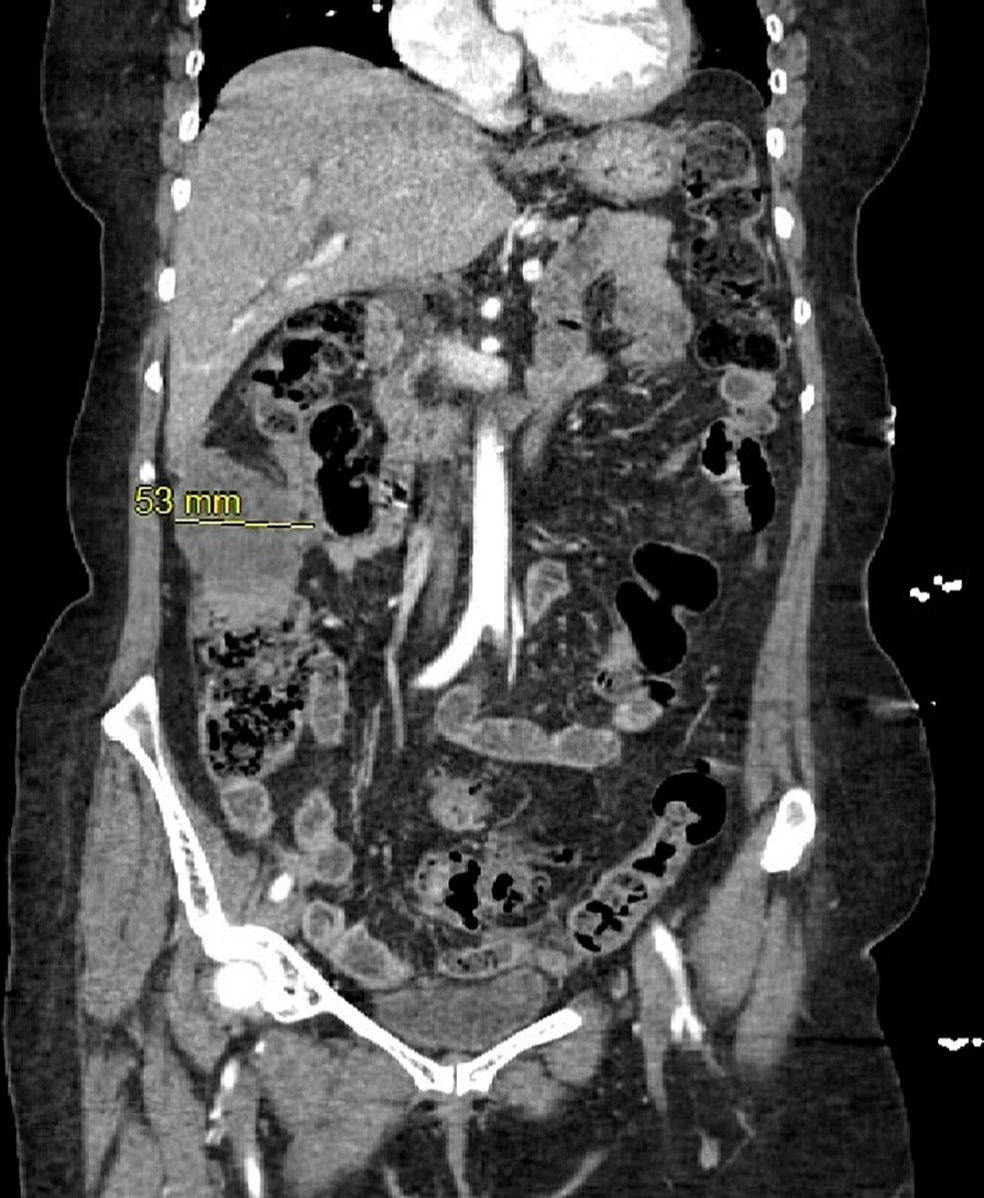
Computed tomography (9CT) abdomen Yellow arrow indication 5.3 cm mass

A 70-year-old female presented to the emergency room complaining of RUQ abdominal pain, nausea, and malaise for several days. She also reported significant weight loss for a couple of months. As a part of her diagnostic workup, computed tomography (CT) abdomen showed a large mass in the region of the hepatic flexure and ascending colon, which was contiguous to a 5-centimeter (cm) mass in the lower portion of segment VI of the liver (Figure 1).

Also, a smaller mass measuring 2.5 cm in size in the left lobe of the liver was visible on the CT abdomen. Preoperative laboratory work-up including carcinoembryonic antigen and liver function test findings were within normal limits. A colonoscopy was performed, which demonstrated an extrinsic mass, without any mucosal lesion, in the region of the upper ascending colon and hepatic flexure.

**Figure 2:**
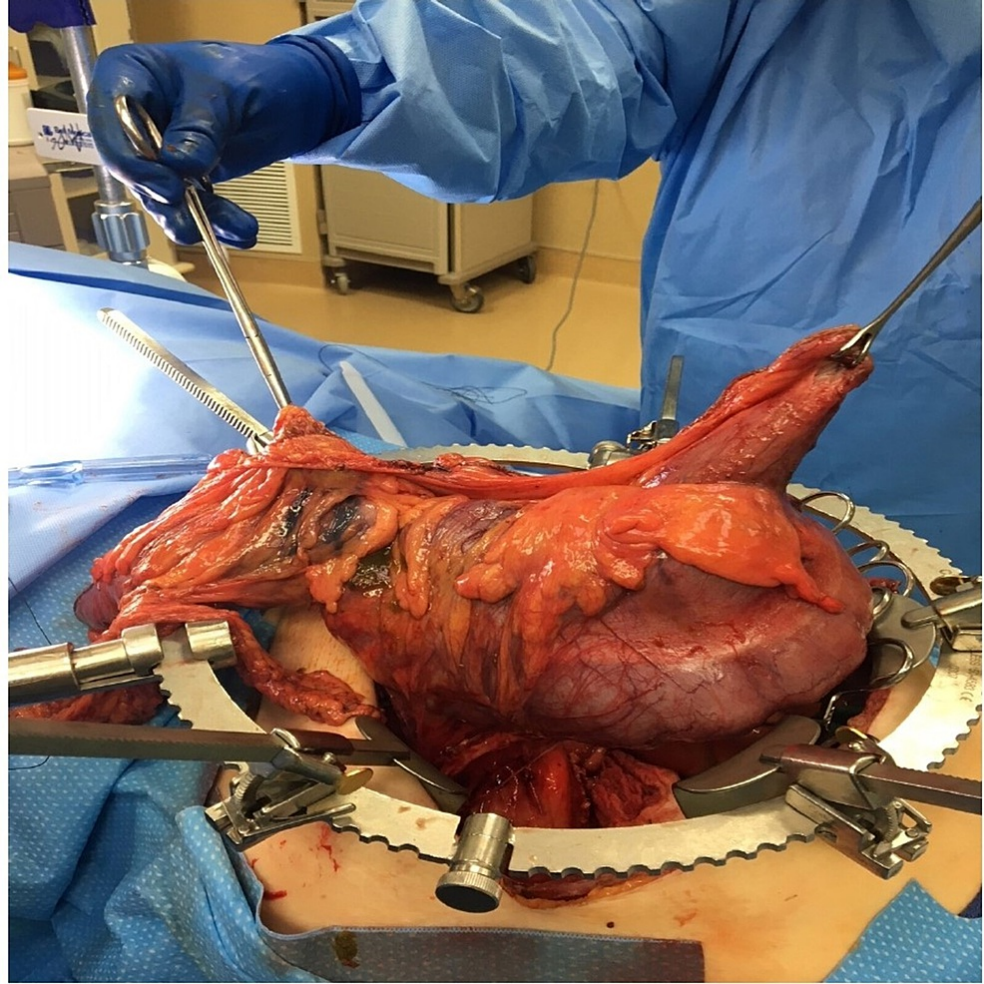
Surgical resection of the mass Tumor en bloc resection of mass with right colon and segment VI of liver

Subsequently, the decision was made to proceed with the surgical exploration of the lesion. A right transverse abdominal incision was made several centimeters below the right costal margin, and a large right colon mass was identified. Next, the terminal ileum was divided with a stapler. High ligation of the right colonic mesentery was then performed using suture ligatures. The transverse colon was divided with a stapler to the right of the middle colic artery. The transverse mesentery was taken to join the mesenteric cut from the distal right colon. In order to take this tumor en bloc, segment VI of the liver was taken down using electrocautery, compression, clipping, and suture ligation of vessels. This en bloc resection was then mobilized from the retroperitoneum, taking the involved Gerota fat off the kidney, providing a complete resection (Figure 2).

**Figure 3:**
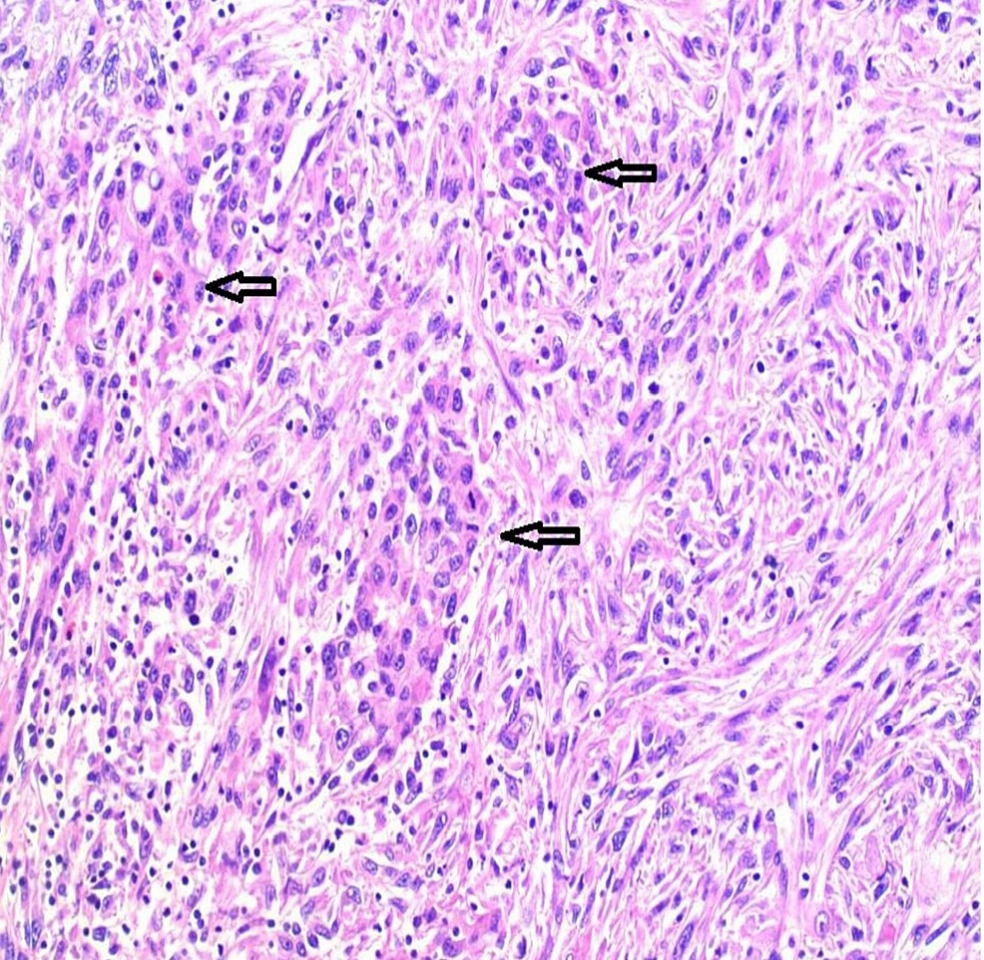
Histological confirmation of the malignant peritoneal mesothelioma Malignant peritoneal mesothelioma - biphasic component. The spindle cell component merges with the epithelioid component (arrows), giving the classification of biphasic variant.

Next, a primary anastomosis was performed between the terminal ileum and the left transverse colon with a stapler, and the suture line was reinforced with interrupted 3-0 silk lambert sutures. The wound was closed in layers using single strand #1 PDS*II. Scarpa’s fascia was closed with an interrupted 3-0 Vicryl suture, and the skin was closed with a running intradermal 3-0 Monocryl suture. The surgical specimens retrieved were sent for histological and immunological examinations, which resulted in postoperative pathologic diagnostic of BPM (Figure 3).

Initially, the postoperative course was complicated by failure to thrive. Total parenteral nutrition was initiated on postoperative day (POD) 3. She had a return of bowel function on POD 6. Following a high leukocytosis of 23.8, an infra-hepatic fluid collection was identified on a CT scan on POD 11. Subsequently, CT-guided drainage was performed, and 15 ccs of bilious fluid were aspirated upon placement of the catheter. On POD 14, her leukocytosis had normalized to 10.1. Following an increase in oral intake, she was cleared for discharge on POD 15. The patient died several weeks later, likely related to the highly aggressive nature of the disease.

## Discussion

Of the 3,300 mesothelioma cases per year, less than 20% are peritoneal, with approximately two times as many males as females [4]. Exposure to a hazardous material is highly associated with mesothelioma but was not closely regulated until the Occupational Safety and Health Administration's Occupational Safety and Health Act of 1970, which ensured environments were free of recognized hazardous material [6]. The literature describes the most commonly affected age group as individuals in their sixth to seventh decade of life who were likely exposed to these environments before the 1970s [7]. These individuals carry a higher risk of association with asbestos; however, they have also been linked to other risk factors such as radiation exposure, thorium, talc, erionite, mica exposure, history of Mediterranean fever, or diffuse lymphocytic lymphoma [8].

Despite various risk exposures, survival depends on three basic histological patterns: epithelioid type (most frequent and better prognosis), sarcomatoid (most rare and worst prognosis), and biphasic (combination of the two) [6]. Our patient was a female who was slightly above the average age of presentation at 70 years old. She was originally from Puerto Rico, and exposure risk was unknown to her family.

BPM is defined as epithelioid based, with at least 10% of the composition being a sarcomatoid pattern [9]. Literature reports a direct correlation to decreased survival with the increased presence of sarcomatoid patterns [10]. BPM comprises less than 25% of cases and is rarely reported in the literature [7]. Similarly, literature has yet to identify specific signs and symptoms associated with PM, which leads to a delayed diagnosis on an average of 122 days from the onset of symptoms [11]. Most patients will present with some degree of abdominal pain and distension along with ascites, intestinal obstruction, hypercoagulability, fever, or other symptoms associated with malignancy such as weight loss, anorexia, or fatigue [4]. Our patient presented with similarly nonspecific symptoms of RUQ abdominal pain, malaise for several days, nausea, as well as weight loss for a couple of months.

Moreover, diagnosis may be further delayed if a thorough evaluation is not performed. Although no imaging modality has proven to be sensitive or specific for diagnosing PM, CT abdomen and pelvis (CTAP) with IV contrast, as was used in our patient, provides essential information [1,3]. The common findings on CT are masses without ascites, ascites, and nodules, or mixed [12]. Our patient's CTAP included a large group visualized in the hepatic flexure and ascending colon contiguous to a 5 cm mass in the lower portion of segment VI of the liver and a smaller mass measuring 2.5 cm in size visualized in the left lower lobe of the liver. In addition to radiological findings, immunohistochemical stains provide an accurate diagnosis. The standard of diagnosing PM would be tissue biopsy via ultrasound, CT, or laparoscopic guidance [12]. As was the case for our patient, the stage of PM is typically advanced at the time of diagnosis, making it challenging to provide the proper treatment.

Despite the poor prognosis, radical resection remains the mainstay of treatment for PM. The combination of CRS and HIPEC is found to extend survival to an average of 36 months [3,6,7,9,11]. Other treatments include a variety of chemotherapy and platinum agents and immunotherapy [1-5,8,13-15]. At the time of our patient’s radical resection, the diagnosis was unclear. Due to the late diagnosis of our case, CRS, HIPEC, and chemotherapy/platinum agents were not an option. Despite efforts to find treatment, BPM remains a lethal disease with our patients.

## Conclusions

BPM is a rare disease that is difficult to diagnose or treat. The average survival of untreated peritoneal mesothelioma is six months. Radical resection, CRS, and HIPEC improve survival; however, the disease is eventually fatal. To provide a better understanding of the pathogenesis and factors impacting survival, all patients diagnosed with BPM should be enrolled in a large-scale registry nationwide.

## References

[1] Brustugun OT, Nilssen Y, Eide IJ (2021). Epidemiology and outcome of peritoneal and pleural mesothelioma subtypes in Norway. A 20 year nation-wide study. Acta Oncol.

[2] Atre ID, Watane GV, Harisinghani MG (2021). Malignant peritoneal mesothelioma: correlation between CT imaging features and histologic subtypes. Abdom Radiol (NY).

[3] Cunha P, Luz Z, Sees I (2002). Malignant peritoneal mesothelioma -- diagnostic and therapeutic difficulties (Article in Portuguese). Acta Med Port.

[4] Raptopoulos V (1985). Peritoneal mesothelioma. Crit Rev Diagn Imaging.

[5] Bridda A, Padoan I, Mencarelli R, Frego M (2007). Peritoneal mesothelioma: a review. MedGenMed.

[6] Fujishima F, Konosu-Fukaya S, Nabeshima K (2021). Histological and immunohistochemical characteristics and p16 status studied by FISH in six incidentally detected cases of well-differentiated papillary mesothelioma of the peritoneum. Indian J Pathol Microbiol.

[7] Sato T, Nakanishi H, Akao K, Okuda M, Mukai S, Kiyono T, Sekido Y (2021). Three newly established immortalized mesothelial cell lines exhibit morphological phenotypes corresponding to malignant mesothelioma epithelioid, intermediate, and sarcomatoid types, respectively. Cancer Cell Int.

[8] Yu Y, Li XB, Lin YL (2021). Efficacy of 1 384 cases of peritoneal carcinomatosis underwent cytoreductive surgery plus hyperthermic intraperitoneal chemotherapy (Article in Chinese). Zhonghua Wei Chang Wai Ke Za Zhi.

[9] Ghafoor A, Mian I, Wagner C (2021). Phase 2 study of olaparib in malignant mesothelioma and correlation of efficacy with germline or somatic mutations in BAP1 gene. JTO Clin Res Rep.

[10] Malekzadeh P, Good M, Hughes MS (2021). Cytoreductive surgery and hyperthermic intraperitoneal chemotherapy (HIPEC) with cisplatin in pediatric patients with peritoneal mesothelioma: a single institution experience and long term follow up. Int J Hyperthermia.

[11] Sugarbaker PH, Chang D, Jelinek JS (2021). Concerning CT features predict outcome of treatment in patients with malignant peritoneal mesothelioma. Eur J Surg Oncol.

[12] Marchevsky AM, LeStang N, Hiroshima K (2017). The differential diagnosis between pleural sarcomatoid mesothelioma and spindle cell/pleomorphic (sarcomatoid) carcinomas of the lung: evidence-based guidelines from the International Mesothelioma Panel and the MESOPATH National Reference Center. Hum Pathol.

[13] Ahmed I, Tipu SA, Ishtiaq S (2013). Malignant mesothelioma. Pak J Med Sci.

[14] Mujahed T, Tazelaar HD, Sukov WR (2021). Malignant peritoneal mesothelioma arising in young adults with long-standing indwelling intra-abdominal shunt catheters. Am J Surg Pathol.

[15] Ullah A, Waheed A, Khan J (2022). Incidence, survival analysis and future perspective of primary peritoneal mesothelioma (PPM): a population-based study from SEER database. Cancers.

