# Correction: Biphasic Peritoneal Mesothelioma: A Lethal Clinical Entity

**DOI:** 10.7759/cureus.c120

**Published:** 2023-05-30

**Authors:** Jill David, Abdul Waheed, Shahin Foroutan, Audrey McCloskey, Harmanprit Randhawa, Frederick D Cason

**Affiliations:** 1 General Surgery, HCA Healthcare/University of South Florida Morsani College of Medicine GME/Regional Medical Center Bayonet Point Program, Hudson, Florida, USA; 2 Surgery, San Jaoquin General Hospital, San Jaoquin, USA; 3 General Surgery/Trauma and Critical Care, San Joaquin General Hospital, French Camp, USA; 4 Surgery, St. George's University School of Medicine, St. Georges, GRD; 5 Emergency Medicine, San Joaquin General Hospital, French Camp, USA; 6 Surgery, San Joaquin General Hospital, French Camp, USA

This article has been corrected at the request of the authors to add Jill David as first author. Due to some confusion among the authors, Jill David was originally omitted from the author list. She contacted the journal upon becoming aware of this and the authors agreed that this was an oversight.

